# Prevalence and Risk Factors of Mental Health Symptoms and Suicidal Behavior Among University Students in Wuhan, China During the COVID-19 Pandemic

**DOI:** 10.3389/fpsyt.2021.695017

**Published:** 2021-07-13

**Authors:** Yingying Xu, Sizhen Su, Zhendong Jiang, Suihuai Guo, Qingdong Lu, Lin Liu, Yimiao Zhao, Ping Wu, Jianyu Que, Le Shi, Jiahui Deng, Shiqiu Meng, Wei Yan, Yankun Sun, Kai Yuan, Xiao Lin, Siwei Sun, Arun V. Ravindran, Sijing Chen, Yun Kwok Wing, Xiangdong Tang, Maosheng Ran, Yu Lu, Jie Shi, Guofu Huang, Yanping Bao, Lin Lu

**Affiliations:** ^1^National Institute on Drug Dependence and Beijing Key Laboratory of Drug Dependence, Peking University, Beijing, China; ^2^School of Public Health, Peking University, Beijing, China; ^3^Peking University Sixth Hospital, Peking University Institute of Mental Health, NHC Key Laboratory of Mental Health (Peking University), National Clinical Research Center for Mental Disorders (Peking University Sixth Hospital), Beijing, China; ^4^Wuhan Wuchang Hospital, Wuhan University of Science and Technology, Wuhan, China; ^5^Department of Psychiatry, University of Toronto, Toronto, ON, Canada; ^6^Department of Psychiatry, Faculty of Medicine, Chinese University of Hong Kong, Hong Kong SAR, China; ^7^Sleep Medicine Center, Department of Respiratory and Critical Care Medicine, Mental Health Center and Translational Neuroscience Center, State Key Laboratory of Biotherapy, West China Hospital, Sichuan University, Chengdu, China; ^8^Department of Social Work and Social Administration, University of Hong Kong, Hong Kong, China; ^9^Peking-Tsinghua Center for Life Sciences and PKU-IDG/McGovern Institute for Brain Research, Peking University, Beijing, China

**Keywords:** university students, COVID-19, prevalence, mental health, suicidal behavior

## Abstract

**Background:** University students who are exposed to coronavirus disease 2019 (COVID-19) could be mentally distressed. We aimed to evaluate the pattern and risk factors of mental health and suicidal behavior among students who experienced long-term school closure due to the COVID-19 pandemic.

**Methods:** This large-sample, cross-sectional, online survey was conducted from June 29, 2020, to July 18, 2020. Eleven thousand two hundred fifty four participants were recruited from 30 universities located in Wuhan, Hubei Province, China. The prevalence of symptoms of depression, anxiety, insomnia, and posttraumatic stress disorder (PTSD) and suicidal behavior was evaluated using the Patient Health Questionnaire-9, Generalized Anxiety Disorder-7, Insomnia Severity Index, Posttraumatic Stress Disorder Checklist for DSM-5, and questions about suicidal ideation and attempts, respectively. Logistic regression was used to explore risk factors for mental health problems and suicidal behavior.

**Results:** The prevalence of mental health problems was 41.5% for depressive symptoms, 32.6% for anxiety symptoms, 35.0% for insomnia symptoms, 8.5% for PTSD symptoms, and 2.0% for suicidal behavior. Participants with high stress during the pandemic were at higher risk of symptoms of depression [adjusted odds ratio (OR) = 1.67, 95% confidence interval (CI) = 1.43–1.95, *p* < 0.01), anxiety (adjusted OR = 1.90, 95% CI = 1.63–2.23, *p* < 0.01), insomnia (adjusted OR = 1.64, 95% CI = 1.44–1.87, *p* < 0.01), PTSD (adjusted OR = 1.71, 95% CI = 1.38–2.11, *p* < 0.01) and suicidal behavior (adjusted OR = 3.51, 95% CI = 2.28–5.40, *p* < 0.01). Distant relationship with parents, changes in lifestyle and alcohol use during the pandemic were associated with higher risk of mental health symptoms and suicidal behavior, whereas regular physical exercise reduced the risk of mental health problems.

**Conclusions:** The psychological symptoms and suicidal behavior were relatively high among students who attended university in Wuhan, China after 6 months of the COVID-19 outbreak in China. Comprehensive mental health services and suicide prevention strategies are essential for university students during the COVID-19 pandemic.

## Introduction

In December 2019, the coronavirus disease 2019 (COVID-19) outbreak began and was officially announced as a pandemic by the World Health Organization (1). People's lifestyles were profoundly changed by the serious health outcomes of COVID-19, and extremely strict containment measures were taken, including lockdown, quarantine, school closures, and social distancing (2, 3). The unpredictability and uncertainty of the COVID-19 pandemic that are associated with containment strategies and financial loss are among the major stressors that contribute to widespread emotional distress and a higher risk of psychiatric problems in vulnerable populations worldwide (4, 5), including COVID-19-infected cases (6), healthcare workers (7, 8), the elderly (9), and children and adolescents (10).

Mental health status and suicidal behavior among university students that are exacerbated due to long-term mandatory school closures during the pandemic require specific attention. University students might struggle with loneliness and isolation and experience severe psychological distress during the pandemic because of disconnections from friends and partners (11). Students who attended University in an epidemic area during the COVID-19 outbreak might suffer from stigma and discrimination, which were associated with a higher risk of mental health problems (2, 12). Additionally, mental health problem was strongly associated with suicidal ideation and attempts (13, 14). Individuals who had received counseling services on campus could no longer access counseling services, which may exacerbate their mental well-being and increase their risk of substance abuse or even suicidal behavior (11). Suicide is the second leading cause of death among individuals aged 15-29 years globally (15). A meta-analysis showed that the pooled prevalence of lifetime suicidal ideation and attempts among college students was 22.3 and 3.2%, respectively (16).

Several studies reported a potential rise of mental health problems and suicidal behavior among university students during the pandemic (2, 17–19). Major studies focused on a specific mental health problem and had a small sample size (20, 21), evaluated only specific groups (e.g., medical students) (19), or evaluated only one University (18). An exception was the online survey which assessed the prevalence of suicidal ideation, stress, and other mental symptoms among 69,054 university students in France (22). However, most of these investigations including this survey of France were conducted during early stages of the pandemic (18, 23). To date, many countries are facing substitantial threats from the ongoing pandemic and long-term quarantine. University students leaved school and have been changing their normal study habits and lifestyle for a prolonged period of time. Understanding their mental health status and related risk factors is vital for improving mental health, the development of public response strategies, and reopening schools in the future.

To better evaluate the impact of the long-term COVID-19 pandemic on psychological status and suicidal behavior among university students who experienced isolation due to returning home from Wuhan in early days of the pandemic, especially after long-term quarantine and school closure. We conducted a cross-sectional online survey to investigate the prevalence of mental health symptoms and suicidal behavior and potential risk factors among university students 6 months after the COVID-19 pandemic began in China.

## Methods

### Study Design

The present study followed the American Association for Public Opinion Research reporting guidelines. Approval from the ethics committee of Peking University Sixth Hospital (Institute of Mental Health) was received before the study began. Informed consent was received online before the respondents began the questionnaire.

This cross-sectional online survey was conducted from June 29, 2020, to July 18, 2020. Based on convenience sampling method, in order to make our sample more representative, the survey involved universities including key universities, ordinary universities and vocational and technical colleges, and the types of subjects of universities were also taken into account. Finally, university students were recruited from 30 universities located in Wuhan, Hubei Province, China (24).

Self-administered questionnaires were sent to students through an online platform and all University classes level were invited to fill the questionaire. Before the survey began, the details of the survey were given by the class instructor to the class wechat group of students or psychological teacher, the students take part in the survey voluntarily and all of the participants, who then provided informed consent electronically. The investigation was anonymous, and the confidentiality of all information was ensured.

### Study Population

A total of 65,845 students clicked on the survey link, and 11,325 individuals provided the informed consent, for a participation rate of 17.20%. The respondents came from 31 province-level regions in China and attended 30 universities in Wuhan, Hubei Province, China. They returned home from Wuhan for winter vacation when the COVID-19 outbreak began and experienced quarantine in their own home during the pandemic. A total of 11,325 individuals provided informed consent and completed the questionnaires, among whom 71 individuals were excluded because of invalid questionnaire. Participants were excluded if their Body Mass Index (BMI) were out of the range of 13–50, younger than 15 years, or contradictory options about the same questions, for example, their marital status was married while they were younger than 20 years or the type of student they fill in wasn't consistent with that their school enrolled.

### Measurements and Covariates

The primary outcome was the prevalence and associated factors of symptoms of depression, anxiety, insomnia and PTSD and suicidal behavior. The survey lasted approximately 10 min. The first part gathered demographic information, including gender, age, nationality, province, city/town, level of education, accommodation in the school, satisfaction with major, academic performance, graduates, peer and teacher relationships, relationship with immediate family, and so on. The second part included questions about the pandemic. The third part focused on the individuals' frequent behaviors before and during the pandemic. The three parts of the survey are included in the Supplementary Table 1.

The fourth part of the questionnaire focused on mental health, including a family history of mental illness, questions about suicidal behavior, and validated measurement tools (in Chinese). We used the 9-item Patient Health Questionnaire (PHQ-9) (25), Generalized Anxiety Disorder-7 (GAD-7) (26), Insomnia Severity Index (ISI) (27), and Posttraumatic Stress Disorder Checklist for DSM-5 (PCL-5) (28) to assess symptoms of depression, anxiety, insomnia, and PTSD, respectively. Total scores on these scales were interpreted as the following: PHQ-9 (normal, 0–4; mild depression, 5–9; moderate depression, 10–14; severe depression, 15–27), GAD-7 (normal, 0–4; mild anxiety, 5–9; moderate anxiety, 10–14; severe anxiety, 15–21), ISI (normal, 0–7; subthreshold insomnia, 8–14; moderate insomnia, 15–21; severe insomnia, 22–28), PCL-5 (normal, 0–32; positive for PTSD, 33–80). The cutoff scores for detecting risk factors of symptoms of depression, anxiety, insomnia, and PTSD were 5, 5, 8, and 33, respectively. Suicidal behavior during lifetime included active suicidal ideation and suicide attempts, which were initially screened using a modified inventory (29) in this study. Participants who answered in the affirmative were asked about the occurrence of suicidal ideation and suicide attempts specifically after the COVID-19 outbreak [e.g., “I thought about killing myself after the COVID-19 outbreak” [Yes/no]; “I deliberately tried to kill myself after the COVID-19 outbreak” (Yes/no)].

### Statistical Analysis

Descriptive statistics were used to present demographic data. χ^2^ tests were used to compare the prevalence of mild-to-severe mental health symptoms and suicidal ideation and attempts and multiple comparison corrections were conducted for χ^2^ tests with more than 2 groups or 2 categories. To explore the factors that were potentially associated with depression, anxiety, insomnia, PTSD symptoms, and suicidal behavior, multiple logistic regression analyses were performed, and odds ratios (ORs) and 95% confidence intervals (CIs) are presented. All variables that were statistically significant in the unadjusted regression analysis were entered into the multivariable model, and then the backward method was applied to determine the variables that were statistically significant in the multivariable analysis. The variance inflation factor (VIF) of these variables >10 indicates high collinearity (30). All of the data analyses were performed using SPSS 22 software. The level of significance was set to *p* < 0.05, and all tests were two-tailed.

## Results

### Demographic Characteristics

A total of 11,254 eligible participants from 31 province-level regions in China were included in the final analysis. Of the total sample, 4,054 individuals (36.02%) were male, 8,139 individuals (72.32%) were 15–20 years old, 408 individuals (3.63%) were graduates. More than half of the respondents [6,960 (61.84%)] lived in Hubei province during the pandemic, and 1,402 (12.46%) lived in Wuhan. Of the total number, 6,455 (57.36%) had a harmonious relationship with their classmates and teachers, and most of them had a close relationship with their parents [7,797 (69.28%)]. Furthermore, 76 individuals (0.68%) had confirmed or suspected COVID-19 or were in close contact with confirmed cases, 2,235 individuals (19.86%) were under high stress, and 8,279 individuals (73.56%) experienced changes in lifestyle during the pandemic. A total of 663 individuals (5.89%) reported alcohol use during the pandemic, and 424 individuals (3.77%) reported tobacco use during the pandemic. Additional demographic pandemic-related characteristics are presented in Table 1.

**Table 1:** Descriptive statistics of demographic characteristics and pandemic-related information for students.

**Factor**	**Participants (no. [%])**
Overall	11,254 (100.00)
**Gender**	
Female	7,200 (63.98)
Male	4,054 (36.02)
**Age (years)**	
15–20	8,139 (72.32)
>20	3,115 (27.68)
**Nationality**	
Han	10,421 (92.60)
National minority	833 (7.40)
**Universities**	
Key universities	2,964 (26.34)
Ordinary universities	6,090 (54.11)
Vocational and technical colleges	2,200 (19.55)
**Level of education**	
Less than college	3,209 (28.51)
College degree or higher	8,005 (71.13)
Postgraduate	40 (0.36)
**Living area**	
Wuhan	1,402 (12.46)
Hubei Province outside Wuhan	5,558 (49.39)
Other provinces	4,294 (38.16)
**Are you graduates?**	
Yes	408 (3.63)
No	10,846 (96.37)
**Relationship with classmates and teachers**	
Strained relationship	41 (0.36)
General relationship	4,758 (42.28)
Harmonious relationship	6,455 (57.36)
**Intimacy with parents**	
Distant	132 (1.17)
General	3,325 (29.55)
Close	7,797 (69.28)
**Positive individual history of mental illness**	
Yes	2,863 (25.44)
No	8,391 (74.56)
**Positive family history of mental illness**	
Yes	377 (3.35)
Unknown	1,608 (14.29)
No	9,269 (82.36)
**Are you infected with COVID-19 (confirmed or suspected cases or close contacts)?**	
Yes	76 (0.68)
No	11,178 (99.32)
**Stress during COVID-19 pandemic**	
High	2,235 (19.86)
Moderate	4,025 (35.77)
Low	4,994 (44.38)
**Changes in lifestyle during COVID-19 pandemic**	
Yes	8,279 (73.56)
No	2,975 (26.44)
**Alcohol use during COVID-19 pandemic**	
Yes	663 (5.89)
No	10,591 (94.11)
**Tobacco use during COVID-19 pandemic**	
Yes	424 (3.77)
No	10,830 (96.23)
**Have you learned about some mental health knowledge during COVID-19 pandemic?**	
Yes	6,746 (59.94)
No	4,508 (40.06)
**Do you engage in regular physical exercise during COVID-19 pandemic?**	
Yes	4,696 (41.73)
No	6,558 (58.27)

### Prevalence of Symptoms of Depression, Anxiety, Insomnia, and PTSD and Suicidal Behavior

In this survey, among the 11,254 university students, 5,931 (52.70%) reported at least one symptom of depression, anxiety, insomnia, or PTSD or suicidal behavior. The prevalence of depressive symptoms among the total sample was 41.52% [4,673, including 2,970 (26.39%) with mild depression and 1,703 (15.13%) with moderate-to-severe depression]. The prevalence of anxiety was 32.58% [3,666, including 2,633 (23.40%) with mild anxiety and 1,033 (9.18%) with moderate-to-severe anxiety]. The prevalence of insomnia was 35.00% [3 939, including 3,181 (28.27%) with subthreshold insomnia and 758 (6.74%) with moderate-to-severe insomnia]. The prevalence of PTSD symptoms was 8.46% (952). The prevalence of suicidal behavior was 2.03% [229, including 218 (1.94%) with suicidal ideation and 11 (0.10%) with suicide attempts].

Table 2 shows the severity of mental health symptoms and the presence of suicidal behavior, stratified by demographic characteristics and COVID-19-related factors. Female university students had a significantly lower prevalence of PTSD symptoms (7.50 vs. 10.16%, *p* < 0.01) but a higher prevalence of suicidal behavior compared with males (2.39 vs. 1.41%, *p* < 0.01). The prevalence of mental health symptoms and suicidal behavior was higher among graduates, and students with strained relationships with their classmates/teachers and parents. Individuals who had a prior history of mental illness and positive family history of psychosis had a higher prevalence of mental health symptoms and suicidal behavior. Participants with confirmed or suspected COVID-19 or were in close contact with confirmed cases had a higher prevalence of mental health symptoms and suicidal behavior. The prevalence of mental health problems and suicidal behavior were higher among university students with high stress (depression: 61.83 vs. 26.75%, *p* < 0.01; anxiety: 52.44 vs. 18.46%, *p* < 0.01; insomnia: 52.89 vs. 22.63%, *p* < 0.01; PTSD: 19.37 vs. 3.08%, *p* < 0.01; suicidal behavior: 5.46 vs. 0.58%, *p* < 0.01).

**Table 2:** Prevalence of symptoms of depression, anxiety, insomnia, and PTSD and suicidal behavior in the students.

	**Depression^a^**				**Anxiety^b^**				**Insomnia^c^**				**PTSD^d^**			**Suicidal behavior**	
	**Participants (no. [%])**				**Participants (no. [%])**				**Participants (no. [%])**				**Participants (no. [%])**			**Participants (no. [%])**	
**Variable**	**Normal**	**Mild**	**Moderate to severe**	***P-*value^e^**	**Normal**	**Mild**	**Moderate to severe**	***P-*value^e^**	**Normal**	**Subthreshold**	**Moderate to severe**	***P-*value^e^**	**Normal**	**Positive**	***p* value**		***p* value**
Overall	6,581 (58.48)	2,970 (26.39)	1,703 (15.13)		7,588 (67.42)	2,633 (23.40)	1,033 (9.18)		7,315 (65.00)	3,181 (28.27)	758 (6.74)		10,302 (91.54)	952 (8.46)		229 (2.03)	
**Gender**																	
Female	4,122 (57.25)	1,978 (27.47)	1,100 (15.28)	<0.01	4,802 (66.69)	1,745 (24.24)	653 (9.07)	0.03	4,676 (64.94)	2,050 (28.47)	474 (6.58)	0.87	6,660 (92.50)	540 (7.50)	<0.01	172 (2.39)	<0.01
Male	2,459 (60.66)	992 (24.47)	603 (14.87)		2,786 (68.72)	888 (21.90)	380 (9.37)		2,639 (65.10)	1,131 (27.90)	284 (7.01)		3,642 (89.84)	412 (10.16)		57 (1.41)	
**Living area^#^**																	
Wuhan	839 (59.84)	340 (24.25)	223 (15.91)	0.28	956 (68.19)	303 (21.61)	143 (10.20)	0.23	911 (64.98)	385 (27.46)	106 (7.56)	0.42	1,258 (89.73)	144 (10.27)	0.04	41 (2.92)	0.02
Hubei Province outside Wuhan	3,243 (58.35)	1,520 (27.35)	795 (14.30)	0.88	3,779 (67.99)	1,305 (23.48)	474 (8.53)	0.10	3,665 (65.94)	1,552 (27.92)	341 (6.14)	0.03	5,113 (91.99)	445 (8.01)	0.42	106 (1.91)	0.99
Other provinces	2,499 (58.20)	1,110 (25.85)	685 (15.95)		2,853 (66.44)	1,025 (23.87)	416 (9.69)		2,739 (63.79)	1,244 (28.97)	311 (7.24)		3,931 (91.55)	363 (8.45)		82 (1.91)	
**Are you graduates?**																	
Yes	226 (55.39)	99 (24.26)	83 (20.34)	0.20	263 (64.46)	84 (20.59)	61 (14.95)	0.19	243 (59.56)	124 (30.39)	41 (10.05)	0.02	340 (83.33)	68 (16.67)	<0.01	17 (4.17)	<0.01
No	6,355 (58.59)	2,871 (26.47)	1,620 (14.94)		7,325 (67.54)	2,549 (23.50)	972 (8.96)		7,072 (65.20)	3,057 (28.19)	717 (6.61)		9,962 (91.85)	884 (8.15)		212 (1.95)	
**Relationship with classmates and teachers^#^**																	
Strained relationship	12 (29.27)	9 (21.95)	20 (48.78)	<0.01	18 (43.90)	11 (26.83)	12 (29.27)	<0.01	12 (29.27)	16 (39.02)	13 (31.71)	<0.01	27 (65.85)	14 (34.15)	<0.01	6 (14.63)	<0.01
General relationship	2,304 (48.42)	1,482 (31.15)	972 (20.43)	<0.01	2,804 (58.93)	1,383 (29.07)	571 (12.00)	<0.01	2,733 (57.44)	1,613 (33.90)	412 (8.66)	<0.01	4,230 (88.90)	528 (11.10)	<0.01	126 (2.65)	<0.01
Harmonious relationship	4,265 (66.07)	1,479 (22.91)	711 (11.01)		4,766 (73.83)	1,239 (19.19)	450 (6.97)		4,570 (70.80)	1,552 (24.04)	333 (5.16)		6,045 (93.65)	410 (6.35)		97 (1.50)	
**Intimacy with parents^#^**																	
Distant	37 (28.03)	36 (27.27)	59 (44.70)	<0.01	48 (36.36)	41 (31.06)	43 (32.58)	<0.01	46 (34.85)	52 (39.39)	34 (25.76)	<0.01	91 (68.94)	41 (31.06)	<0.01	17 (12.88)	<0.01
General	1,461 (43.94)	1,041 (31.31)	823 (24.75)	<0.01	1,813 (54.53)	1,025 (30.83)	487 (14.65)	<0.01	1,780 (53.53)	1,208 (36.33)	337 (10.14)	<0.01	2,857 (85.92)	468 (14.08)	<0.01	128 (3.85)	<0.01
Close	5,083 (65.19)	1,893 (24.28)	821 (10.53)		5,727 (73.45)	1,567 (20.10)	503 (6.45)		5,489 (70.40)	1,921 (24.64)	387 (4.96)		7,354 (94.32)	443 (5.68)		84 (1.08)	
**Positive individual history of mental illness**																	
Yes	915 (31.96)	1,033 (36.08)	915 (31.96)	<0.01	1,229 (42.93)	1,017 (35.52)	617 (21.55)	<0.01	1,130 (39.47)	1,248 (43.59)	485 (16.94)	<0.01	2,316 (80.89)	547 (19.11)	<0.01	143 (4.99)	<0.01
No	5,666 (67.52)	1,937 (23.08)	788 (9.39)		6,359 (75.78)	1,616 (19.26)	416 (4.96)		6,185 (73.71)	1,933 (23.04)	273 (3.25)		7,986 (95.17)	405 (4.83)		86 (1.02)	
**Positive family history of mental illness^#^**																	
Yes	155 (41.11)	117 (31.03)	105 (27.85)	<0.01	180 (47.75)	124 (32.89)	73 (19.36)	<0.01	196 (51.99)	130 (34.48)	51 (13.53)	<0.01	296 (78.51)	81 (21.49)	<0.01	17 (4.51)	<0.01
Unknown	663 (41.23)	541 (33.64)	404 (25.12)	<0.01	848 (52.74)	491 (30.53)	269 (16.73)	<0.01	866 (53.86)	565 (35.14)	177 (11.01)	<0.01	1,367 (85.01)	241 (14.99)	<0.01	57 (3.54)	<0.01
No	5,763 (62.17)	2,312 (24.94)	1,194 (12.88)		6,560 (70.77)	2,018 (21.77)	691 (7.45)		6,253 (67.46)	2,486 (26.82)	530 (5.72)		8,639 (93.20)	630 (6.80)		155 (1.67)	
**Are you infected with COVID-19** **(confirmed or suspected cases or close contacts)?**																	
Yes	21 (27.63)	18 (23.68)	37 (48.68)	<0.01	25 (32.89)	24 (31.58)	27 (35.53)	<0.01	26 (34.21)	27 (35.53)	23 (30.26)	<0.01	48 (63.16)	28 (36.84)	<0.01	5 (6.58)	0.02
No	6,560 (58.69)	2,952 (26.41)	1,666 (14.90)		7,563 (67.66)	2,609 (23.34)	1,006 (9.00)		7,289 (65.21)	3,154 (28.22)	735 (6.58)		10,254 (91.73)	924 (8.27)		224 (2.00)	
**Stress during COVID-19 pandemic^#^**																	
High	853 (38.17)	702 (31.41)	680 (30.43)	<0.01	1,063 (47.56)	691 (30.92)	481 (21.52)	<0.01	1,053 (47.11)	848 (37.94)	334 (14.94)	<0.01	1,802 (80.63)	433 (19.37)	<0.01	122 (5.46)	<0.01
Moderate	2,070 (51.43)	1,311 (32.57)	644 (16.00)	<0.01	2,453 (60.94)	1,224 (30.41)	348 (8.65)	<0.01	2,398 (59.58)	1,384 (34.39)	243 (6.04)	<0.01	3,696 (91.83)	329 (8.17)	<0.01	78 (1.94)	<0.01
Low	3,658 (73.25)	957 (19.16)	379 (7.59)		4,072 (81.54)	718 (14.38)	204 (4.08)		3,864 (77.37)	949 (19.00)	181 (3.62)		4,804 (96.20)	190 (3.80)		29 (0.58)	
**Changes in lifestyle during COVID-19 pandemic?**																	
Yes	4,409 (53.26)	2,433 (29.39)	1,437 (17.36)	<0.01	5,224 (63.10)	2,185 (26.39)	870 (10.51)	<0.01	4,988 (60.25)	2,631 (31.78)	660 (7.97)	<0.01	7,477 (90.31)	802 (9.69)	<0.01	200 (2.42)	<0.01
No	2,172 (73.01)	537 (18.05)	266 (8.94)		2,364 (79.46)	448 (15.06)	163 (5.48)		2,327 (78.22)	550 (18.49)	98 (3.29)		2,825 (94.96)	150 (5.04)		29 (0.97)	
**Alcohol use during COVID-19 pandemic**																	
Yes	306 (46.15)	190 (28.66)	167 (25.19)	<0.01	380 (57.32)	161 (24.28)	122 (18.40)	<0.01	355 (53.54)	216 (32.58)	92 (13.88)	<0.01	553 (83.41)	110 (16.59)	<0.01	35 (5.28)	<0.01
No	6,275 (59.25)	2,780 (26.25)	1,536 (14.50)		7,208 (68.06)	2,472 (23.34)	911 (8.60)		6,960 (65.72)	2,965 (28.00)	666 (6.29)		9,749 (92.05)	842 (7.95)		194 (1.83)	
**Tobacco use during COVID-19 pandemic**																	
Yes	206 (48.58)	111 (26.18)	107 (25.24)	<0.01	244 (57.55)	99 (23.35)	81 (19.10)	<0.01	241 (56.84)	124 (29.25)	59 (13.92)	<0.01	349 (82.31)	75 (17.69)	<0.01	16 (3.77)	0.01
No	6,375 (58.86)	2,859 (26.40)	1,596 (14.74)		7,344 (67.81)	2,534 (23.40)	952 (8.79)		7,074 (65.32)	3,057 (28.23)	699 (6.45)		9,953 (91.90)	877 (8.10)		213 (1.97)	
**Have you learned about some mental health knowledge during COVID-19 pandemic?**																	
Yes	4,006 (59.38)	1,802 (26.71)	938 (13.90)	0.02	4,598 (68.16)	1,602 (23.75)	546 (8.09)	0.04	4,439 (65.80)	1,883 (27.91)	424 (6.29)	0.03	6,229 (92.34)	517 (7.66)	<0.01	99 (2.20)	0.32
No	2,575 (57.12)	1,168 (25.91)	765 (16.97)		2,990 (66.33)	1,031 (22.87)	487 (10.80)		2,876 (63.80)	1,298 (28.79)	334 (7.41)		4,073 (90.35)	435 (9.65)		130 (1.93)	
**Do you engage in regular physical exercise during COVID-19 pandemic?**																	
Yes	3,018 (64.27)	1,130 (24.06)	548 (11.67)	<0.01	3,380 (71.98)	970 (20.66)	346 (7.37)	<0.01	3,299 (70.25)	1,157 (24.64)	240 (5.11)	<0.01	4,365 (92.95)	331 (7.05)	<0.01	84 (1.79)	0.12
No	3,563 (54.33)	1,840 (28.06)	1,155 (17.61)		4,208 (64.17)	1,663 (25.36)	687 (10.48)		4,016 (61.24)	2,024 (30.86)	518 (7.90)		5,937 (90.53)	621 (9.47)		145 (2.21)	
**Symptoms of depression**																	
Normal	—	—	—		6,219 (94.50)	346 (5.26)	16 (0.24)	<0.01	5,597 (85.05)	934 (14.19)	50 (0.76)	<0.01	6,550 (99.53)	31 (0.47)	<0.01	28 (0.43)	<0.01
Positive	—	—	—		1,369 (29.30)	2,287 (48.94)	1,017 (21.76)		1,718 (36.76)	2,247 (48.08)	708 (15.15)		3,752 (80.29)	921 (19.71)		201 (4.30)	
**Symptoms of anxiety**																	
Normal	6,219 (81.96)	1,190 (15.68)	179 (2.36)	<0.01	—	—	—		6,103 (80.43)	1,358 (17.90)	127 (1.67)	<0.01	7,549 (99.49)	39 (0.51)	<0.01	46 (0.61)	<0.01
Positive	362 (9.87)	1,780 (48.55)	1,524 (41.57)		—	—	—		1,212 (33.06)	1,823 (49.73)	631 (17.21)		2,753 (75.10)	913 (24.90)		183 (4.99)	
**Symptoms of insomnia**																	
Normal	5,597 (76.51)	1,431 (19.56)	287 (3.92)	<0.01	6,103 (83.43)	1,063 (14.53)	149 (2.04)	<0.01	—	—	—		7,207 (98.52)	108 (1.48)	<0.01	63 (0.86)	<0.01
Positive	984 (24.98)	1,539 (39.07)	1,416 (35.95)		1,485 (37.70)	1,570 (39.86)	884 (22.44)		—	—	—		3,095 (78.57)	844 (21.43)		166 (4.21)	
**Symptoms of PTSD**																	
Normal	6,550 (63.58)	2,809 (27.27)	943 (9.15)	<0.01	7,549 (73.28)	2,356 (22.87)	397 (3.85)	<0.01	7,207 (69.96)	2,690 (26.11)	405 (3.93)	<0.01	—	—		126 (1.22)	<0.01
Positive	31 (3.26)	161 (16.91)	760 (79.83)		39 (4.10)	277 (29.10)	636 (66.81)		108 (11.34)	491 (51.58)	353 (37.08)		—	—		103 (10.82)	
**Suicidal behavior**																	
Normal	6,553 (59.44)	2,899 (26.29)	1,573 (14.27)	<0.01	7,542 (68.41)	2,532 (22.97)	951 (8.63)	<0.01	7,252 (65.78)	3,085 (27.98)	688 (6.24)	<0.01	10,176 (92.30)	849 (7.70)	<0.01	—	
Positive	28 (12.23)	71 (31.00)	130 (56.77)		46 (20.09)	101 (44.10)	82 (35.81)		63 (27.51)	96 (41.92)	70 (30.57)		126 (55.02)	103 (44.98)		—	

a *Scores of 5 to 9 on the PHQ-9 were defined as mild depression, and scores of 10 or higher were defined as moderate-to-severe depression*.

b *Scores of 5 to 9 on the GAD-7 were defined as mild anxiety, and scores of 10 or higher were defined as moderate-to-severe anxiety*.

c *Scores of 8 to 14 on the ISI were defined as subthreshold insomnia, and scores of 15 or higher were defined as moderate-to-severe insomnia*.

d *Scores of 33 or higher on the PCL-5 were defined as PTSD symptoms*.

e *χ2 tests were used to compare the prevalence of mild-to-severe mental health symptoms in different populations*.

# *This means the first two groups are compared to the last group respectively*.

Participants who experienced changes in lifestyle, alcohol use, and tobacco use during the pandemic had a significantly higher prevalence of mental health symptoms and suicidal behavior. Participants who learned about mental health knowledge and exercised regularly during the pandemic had a significantly lower prevalence of mental health symptoms. The detailed information are presented in Table 2.

### Factors Associated With Symptoms of Depression, Anxiety, Insomnia, and PTSD and Suicidal Behavior

The results of the univariate logistic regression analysis of demographic and COVID-19 pandemic-related variables are presented in the Supplementary Table 2. In the multivariable analysis, females were associated with a lower risk of PTSD (adjusted OR = 0.61, 95% CI = 0.51–0.72, *p* < 0.01) but a higher risk of suicidal behavior (adjusted OR = 1.93, 95% CI = 1.39–2.68, *p* < 0.01) compared with males. Participants who were under high stress had a higher risk of mental health symptoms and suicidal behavior (depression: adjusted OR = 1.67, 95% CI = 1.43–1.95, *p* < 0.01; anxiety: adjusted OR = 1.90, 95% CI = 1.63–2.23, *p* < 0.01; insomnia: adjusted OR = 1.64, 95% CI = 1.44–1.87, *p* < 0.01; PTSD: adjusted OR = 1.71, 95% CI = 1.38-2.11, *p* < 0.01; suicidal behavior: adjusted OR = 3.51, 95% CI = 2.28–5.40, *p* < 0.01) during the pandemic than participants who were under low stress. Participants who had symptoms of depression were at higher risk of other mental problems (anxiety: adjusted OR = 19.98, 95% CI = 17.50–22.82, *p* < 0.01; insomnia: adjusted OR = 3.73, 95% CI = 3.31–4.19, *p* < 0.01; PTSD: adjusted OR = 3.91, 95% CI = 2.60–5.88, *p* < 0.01) and suicidal behavior (adjusted OR = 2.58, 95% CI = 1.54–4.33, *p* < 0.01). Students who had symptoms of anxiety were at higher risk of other mental problems (depression: adjusted OR = 19.89, 95% CI = 17.41–22.72, *p* < 0.01; insomnia: adjusted OR = 1.94, 95% CI = 1.72–2.19, *p* < 0.01; PTSD: adjusted OR = 13.26, 95% CI = 9.27–18.97, *p* < 0.01) and suicidal behavior (adjusted OR = 1.66, 95% CI = 1.07–2.59, *p* = 0.02). Students with symptoms of insomnia were at higher risk of other mental problems (depression: adjusted OR = 3.68, 95% CI = 3.26–4.14, *p* < 0.01; anxiety: adjusted OR = 1.98, 95% CI = 1.75–2.23, *p* < 0.01; PTSD: adjusted OR = 4.43, 95% CI = 3.54–5.54, *p* < 0.01). Participants who had symptoms of PTSD were at higher risk of other mental problems (depression: adjusted OR = 3.75, 95% CI = 2.47–5.69, *p* < 0.01; anxiety: adjusted OR = 13.45, 95% CI = 9.37–19.30, *p* < 0.01; insomnia: adjusted OR = 4.36, 95% CI = 3.50–5.45, *p* < 0.01) and suicidal behavior (adjusted OR = 2.71, 95% CI = 1.98–3.71, *p* < 0.01). Participants with suicidal behavior were at higher risk of mental problems (depression: adjusted OR = 2.00, 95% CI = 1.17–3.43, *p* = 0.01; anxiety: adjusted OR = 1.56, 95% CI = 1.01–2.41, *p* = 0.04; PTSD: adjusted OR = 2.84, 95% CI = 2.05–3.93, *p* < 0.01). Students with a prior history of mental illness had a higher risk than students without a prior history of mental illness (depression: adjusted OR = 1.81, 95% CI = 1.59–2.07, *p* < 0.01; anxiety: adjusted OR = 1.52, 95% CI = 1.33–1.73, *p* < 0.01; insomnia: adjusted OR = 2.20, 95% CI = 1.98–2.44, *p* < 0.01; PTSD: adjusted OR = 1.53, 95% CI = 1.30–1.81, *p* < 0.01; suicidal behavior: adjusted OR = 1.94, 95% CI = 1.45–2.61, *p* < 0.01). Participants with a positive family history of psychosis had a significantly higher risk of mental health problems than participants without a family history of psychosis (anxiety: adjusted OR = 1.42, 95% CI = 1.04–1.95, *p* = 0.03; PTSD: adjusted OR = 1.89, 95% CI = 1.36–2.64, *p* < 0.01). Participants who engaged in regular exercise during the pandemic reported a lower risk of symptoms of depression (adjusted OR = 0.86, 95% CI = 0.77–0.97, *p* = 0.01), and insomnia (adjusted OR = 0.82, 95% CI = 0.75–0.91, *p* < 0.01). Participants who experienced changes in lifestyle and alcohol use during the pandemic also had a significantly higher risk of mental health problems and suicidal behavior. Since symptoms of depression, anxiety, insomnia, PTSD and suicidal behavior are possibly correlated, we examined the collinearity in five final models and the VIF indicated that no collinearity existed. The highest VIF was 1.51 (symptoms of anxiety) for depression symptoms, 1.52 (symptoms of depression) for anxiety symptoms, 2.07 (symptoms of anxiety) for insomnia symptoms, 2.10 (symptoms of depression) for PTSD symptoms, 2.07 (symptoms of anxiety) for suicidal behavior in each final model. The detailed results of the multivariable analysis are shown in Table 3.

**Table 3:** Multivariable regression analysis of risk factors associated with mental health symptoms and suicidal behavior.

	**Depression^a^**		**Anxiety^b^**		**Insomnia^c^**		**PTSD^d^**		**Suicidal behavior**	
**Variable**	**AOR (95% CI)**	***P-*value**	**AOR (95% CI)**	***P-*value**	**AOR (95% CI)**	***P-*value**	**AOR (95% CI)**	***P-*value**	**AOR (95% CI)**	***P-*value**
**Gender (ref: male)**										
Female	1.13 (1.00–1.27)	0.05	—		—		0.61 (0.51–0.72)	<0.01	1.93 (1.39–2.68)	<0.01
**Are you graduates? (ref: no)**										
Yes	—		—		—		2.04 (1.42–2.93)	<0.01	1.67 (0.96–2.91)	0.07
**Relationship with classmates and teachers (ref: harmonious)**										
Strained	1.73 (0.64–4.66)	0.28	0.68 (0.26–1.80)	0.44	2.74 (1.16–6.47)	0.02	—		—	
General	1.38 (1.23–1.54)	<0.01	1.20 (1.07–1.35)	<0.01	1.17 (1.06–1.29)	<0.01	—		—	
**Intimacy with parents (ref: close)**										
Distant	1.47 (0.82–2.63)	0.20	1.38 (0.81–2.36)	0.24	1.63 (1.04–2.57)	0.03	2.21 (1.36–3.58)	<0.01	4.71 (2.57–8.64)	<0.01
General	1.31 (1.15–1.48)	<0.01	1.16 (1.03–1.32)	0.02	1.17 (1.06–1.30)	<0.01	1.49 (1.26–1.75)	<0.01	2.00 (1.49–2.68)	<0.01
**Positive individual history of mental illness (ref: no)**										
Yes	1.81 (1.59–2.07)	<0.01	1.52 (1.33–1.73)	<0.01	2.20 (1.98–2.44)	<0.01	1.53 (1.30–1.81)	<0.01	1.94 (1.45–2.61)	<0.01
**Positive family history of mental illness (ref: no)**										
Yes	1.02 (0.74–1.41)	0.88	1.42 (1.04–1.95)	0.03	—		1.89 (1.36–2.64)	<0.01	—	
Unknown	1.61 (1.37–1.89)	<0.01	1.20 (1.02–1.40)	0.02	—		1.38 (1.14–1.66)	<0.01	—	
**Stress during COVID-19 epidemic (ref: low)**										
High	1.67 (1.43–1.95)	<0.01	1.90 (1.63–2.23)	<0.01	1.64 (1.44–1.87)	<0.01	1.71 (1.38–2.11)	<0.01	3.51 (2.28–5.40)	<0.01
Moderate	1.42 (1.26–1.61)	<0.01	1.75 (1.53–2.00)	<0.01	1.44 (1.30–1.61)	<0.01	0.98 (0.79–1.21)	0.84	1.99 (1.28–3.10)	<0.01
**Changes in lifestyle during COVID-19 pandemic (ref: no)**										
Yes	1.40 (1.23–1.60)	<0.01	1.19 (1.03–1.38)	0.02	1.55 (1.37–1.74)	<0.01	—		—	
**Alcohol use during COVID-19 pandemic (ref: no)**										
Yes	1.46 (1.15–1.87)	<0.01	—		1.26 (1.03–1.53)	0.02	—		2.31 (1.53–3.49)	<0.01
**Tobacco use during COVID-19 pandemic (ref: no)**										
Yes	—		—		—		1.34 (0.95–1.88)	0.10	—	
**Have you learned about some mental health knowledge during COVID-19 pandemic? (ref: no)**										
Yes	—		—		—		0.86 (0.73–1.01)	0.06	—	
**Do you engage in regular physical exercise during COVID-19 pandemic? (ref: no)**										
Yes	0.86 (0.77–0.97)	0.01	—		0.82 (0.75–0.91)	<0.01	—		—	
**Symptoms of depression (ref: no)**										
Yes	—		19.98 (17.50–22.82)^f^	<0.01	3.73 (3.31–4.19)^g^	<0.01	3.91 (2.60–5.88)^h^	<0.01	2.58 (1.54–4.33)^i^	<0.01
**Symptoms of anxiety (ref: no)**										
Yes	19.89 (17.41–22.72)^e^	<0.01	—		1.94 (1.72–2.19)^g^	<0.01	13.26 (9.27–18.97)^h^	<0.01	1.66 (1.07–2.59)^i^	0.02
**Symptoms of insomnia (ref: no)**										
Yes	3.68 (3.26–4.14)^e^	<0.01	1.98 (1.75–2.23)^f^	<0.01	—		4.43 (3.54–5.54)^h^	<0.01	—	
**Symptoms of PTSD (ref: no)**										
Yes	3.75 (2.47–5.69)^e^	<0.01	13.45 (9.37–19.30)^f^	<0.01	4.36 (3.50–5.45)^g^	<0.01	—		2.71 (1.98–3.71)^i^	<0.01
**Suicidal behavior (ref: no)**										
Yes	2.00 (1.17–3.43)^e^	0.01	1.56 (1.01–2.41)^f^	0.04	—		2.84 (2.05–3.93)^h^	<0.01	—	

a *Depression was defined as PHQ-9 score ≥ 5*.

b *Anxiety was defined as GAD-7 score ≥ 5*.

c *Insomnia was defined as ISI score ≥ 8*.

d *PTSD was defined as PCL-5 score ≥ 33*.

e *The highest VIF in the model of “depression” is 1.51 (symptoms of anxiety), 1.41 (symptoms of insomnia), 1.29 (symptoms of PTSD), and 1.06 (suicidal behavior)*.

f *The highest VIF in the model of “anxiety” is 1.52 (symptoms of depression), 1.46 (symptoms of insomnia), 1.23 (symptoms of PTSD), and 1.06 (suicidal behavior)*.

g *The highest VIF in the model of “insomnia” is 2.00 (symptoms of depression), 2.07 (symptoms of anxiety), and 1.24 (symptoms of PTSD)*.

h *The highest VIF in the model of “PTSD” is 2.10 (symptoms of depression), 2.01 (symptoms of anxiety), 1.44 (symptoms of insomnia), and 1.04 (suicidal behavior)*.

i *The highest VIF in the model of “suicidal behavior” is 1.98 (symptoms of depression) and 2.07 (symptoms of anxiety), and 1.25 (symptoms of PTSD)*.

## Discussion

The present study investigated the prevalence and factors associated with mental health symptoms and suicidal behavior among university students after long-term quarantine and school closures during the COVID-19 pandemic in China. All of the students in this study had a high risk of COVID-19 exposure because they went home for winter vacation from Wuhan, Hubei Province, where COVID-19 was first identified in China. Compared to other university students in China, they experienced quarantine after returning home and might suffer from stigma and discrimination. Overall, more than half of the participants (52.7%) reported at least one symptom of depression, anxiety, insomnia, or PTSD or suicidal behavior. We identified several vulnerable students and risk factors, including graduates, distant relationships with parents, a past history of mental illness, a positive family history of psychosis, moderate to high stress, changes in lifestyle, and alcohol use during the pandemic. We also found that regular exercise during the pandemic was protective against mental health problems. Altogether, our findings highlight concerns about mental well-being among university students and may contribute to the development of better health policies and population-based long-term mental health management and intervention strategies for future pandemics and other public health emergencies.

The prevalence of anxiety (32.6%) in the present study was higher than the results of a previous epidemiological study (19) among medical college students during the pandemic, showing that 24.9% of participants had symptoms of anxiety. This difference may be attributable to long-term quarantine experience in the present survey and the fact that medical students are a special group who may more easily acquire mental health knowledge. Additionally, with ongoing worries about current academic studies, future employment, personnel relationships, and life stress, university students may be vulnerable to the development of mental and sleep problems (2, 11). The prevalence of symptoms of depression among students in this study was higher than in another online study (31) that was performed 1 month after the COVID-19 outbreak began among undergraduate students, which may be attributable to the impact of long-term quarantine during the pandemic.

However, the prevalence of suicidal ideation (1.9%) in the present study was lower than in a study in which 18.0% of a sample of United States college students reported suicidal thoughts, measured by the PHQ-9 (18). Some previous studies showed that responses to items on the PHQ-9 that included self-harm or passive thoughts of death may not accurately reflect suicidal thoughts compared with questions that are designed specifically to assess suicidality, thus resulting in a higher rate of false positives (32–34). Moreover, the prevalence of suicidal ideation in the present study was lower than in a previous study that was conducted at the peak of the COVID-19 pandemic, which showed that 11.4% of university students reported suicidal thoughts (22). Our survey focused on the late sustained pandemic period, which is, another possible reason for the lower prevalence of suicidal ideation in the present study (35). A previous study showed that suicidality was associated with fear of the infection and the experience of social isolation, which were more serious at the peak of the pandemic than at the late pandemic period (2, 35). Further studies with a longer follow-up period after the COVID-19 pandemic and large geographic coverage are warranted.

The present study identified several risk groups who were more likely to develop mental health problems and suicidal behavior. Females had a significantly higher prevalence of suicidal behavior, which is consistent with the results of another study of senior high school students during the COVID-19 pandemic (36). The present results also showed that strained relationships with classmates/teachers and parents increased the risk of mental health problems. For most students, isolation from social networks was associated with more depressive symptoms (37). Conflicts and tension between family members, which may have increased because of staying at home during the pandemic (38), may descend into domestic violence and lead to a worsening of mental health problems and suicidal behavior (39–41). An interesting finding is that we did not find difference of psychological problems between students from Wuhan/Hubei and that from other provinces in final model. The reason may be that all students in our study attended universities in Wuhan and the finding may also indicated that the mental heath well-bing among university students are needed in whole country, not in special high risk area of COVID-19 pandemic.

Participants who had a prior history of mental health problems reported more severe mental health symptoms and suicidal behavior. A positive history of a psychiatric disorder is highly associated with new-onset mental disorders during a stressful period (42). Additionally, the present study also found that participants with a positive family history of psychosis were more likely to suffer from anxiety, and PTSD. Furthermore, this study showed the issue of coexistent mental health symptoms and suicidal behavior during the pandemic which is consistent with other epidemiologic studies (43–45). Another cross-sectional survey of college students found a strong positive interaction effect between anxiety disorder and depression (44). A large cohort study also showed that 40% individuals who had mental disorders were diagnosed with more than one disorder type (46). Our findings indicated the importance of early detection and intervention of mental health symptoms and suicidal behavior among high risk population. It is necessary to establish mental health promotion strategies, such as health education, early response to public tramatic events to further prevent mental health problems and suicidal behavior during the COVID-19 pandemic (2, 47–49). Furthermore, this study showed that suicidal behavior tends to increase when mental health status is exacerbated during the pandemic, which indicate that the importance of establishing mental health promotion strategies to further prevent suicidal behavior (2, 47, 48). COVID-19 pandemic-related stress, and changes in health-related behaviors (e.g., exercise and alcohol consumption) were associated with a higher risk of mental health problems and suicidal behavior. Participants who were under moderate to high stress had a higher risk of mental health symptoms and suicidal behavior. This is consistent with findings that greater stress was significantly correlated with more negative psychological effects in both patients with severe acute respiratory syndrome and healthy control subjects (50). Previous studies also indicated that people under higher levels of stress, which might have come from work, family, or traumatic life events, were more vulnerable to suicidal behavior compared with control groups (51, 52). Moreover, a higher probability of mental health problems and suicidal behavior was also identified among individuals who experienced changes in lifestyle and who reported alcohol use during the pandemic. As a consequence of school closures, students' daily routines have changed dramatically, such as a reduction of social contacts and an increase of problem internet use, which can result in a higher incidence of mental health problems and suicidal behavior (53, 54). Furthermore, previous studies suggested that alcohol abuse was a major risk factor for mental health problems and suicidal behavior because of increases in impulsive and aggressive behaviors (51, 55–57). Conversely, limiting alcohol consumption might be associated with lower risk of mental illness and suicidal behavior (58). Thus, to avoid mental health problems during the COVID-19 pandemic, young people should maintain a healthy diet and adaptive lifestyle (59). We found that students who engaged in regular physical activities during the pandemic had a lower risk of depression, and insomnia. These findings are consistent with another study (60) of Chinese college students during the pandemic. Similarly, a previous study (22) of university students in France confirmed that more frequent physical activity was associated with less severe self-reported mental health symptoms. Thus, regular exercise is an effective strategy to promote mental well-being during the pandemic.

## Strengths and Limitations

The strengths of this study included university students with high COVID-19 exposure experience in Wuhan, extensive geographic coverage in China, the large sample size, the evaluation of multiple risk factors, and the assessment of risk factors after long-term quarantine. The prevention and control of the pandemic in China is stabilizing, but sporadic cases are still being reported. Thus, issues that are related to prevention and control strategies still need to be addressed. We conducted this study with students who experienced long-term mandatory school closure (i.e., ~one semester of the school year). Students who experience long-term school closure may be more vulnerable than students who experience shorter-term closure (18, 19, 31, 61). The COVID-19 pandemic has persisted for nearly 1 year at the time of this writing, which has negatively impacted the daily lives of people worldwide. Its impact on mental health problems and suicidal behavior need to be explored. Our findings provide useful information for health policies, the identification of at-risk students, and the development of population-specific psychological crisis management.

Our study also has several limitations. First, there were recall bias and information bias due to this survey included some questions about past situations, and selection bias since this was an voluntary online survey and the participation rate is low. Thus, the representativeness of the sample might be insufficient. Second, mental health symptoms and suicidal behavior were based on self-rating scales and items rather than clinical diagnoses. We were also unable to distinguish between preexisting mental health symptoms and new symptoms. Third, this was a cross-sectional study that reflected mental health status and suicidal behavior during the pandemic. Therefore, we were unable to identify associations between mental health problems and risk factors because of unclear chronology. More long-term longitudinal follow-up studies are warranted in the future.

## Conclusions

The prevalence of symptoms of depression, anxiety, insomnia, and PTSD and suicidal behavior was high among university students who attended universities in Wuhan, China after long-term quarantine and school closures, especially among students with high stress, who experienced changes in lifestyle, and who reported alcohol use during the pandemic. Furthermore, graduation, distant relations with parents, and a personal or family history of mental illness were also associated with a higher risk of adverse mental health problems and suicidal behavior. These findings suggest that the COVID-19 pandemic may have severe and negative effects on mental health and suicidal behavior among vulnerable university students. Specific interventions that promote mental well-being among university students who are exposed to the long-term impact of the COVID-19 pandemic should be implemented.

## Data Availability Statement

The raw data supporting the conclusions of this article will be made available by the authors, without undue reservation.

## Ethics Statement

The studies involving human participants were reviewed and approved by The ethics committee of Peking University Sixth Hospital (Institute of Mental Health). Written informed consent from the participants' legal guardian/next of kin was not required to participate in this study in accordance with the national legislation and the institutional requirements.

## Author Contributions

LLu, YB, and GH designed this survey, commented on and revised the manuscript, and wrote the final version. YX and SSu were responsible for data analysis and writing the initial draft. ZJ, QL, SG, YL, GH, and YB contributed to recruiting participants and data collection. QL, LLi, YZ, LS, JQ, JD, SM, WY, YS, PW, KY, XL, SSun, and MR designed the questionnaire and commented on and revised the manuscript. AR, SC, YW, XT, and JS revised the manuscript. All of the authors contributed to the final draft of the manuscript.

## Conflict of Interest

The authors declare that the research was conducted in the absence of any commercial or financial relationships that could be construed as a potential conflict of interest.
