# Supplementary material for: Prevalence and Risk Factors of Mental Health Symptoms and Suicidal Behavior Among University Students in Wuhan, China During the COVID-19 Pandemic
Source: Front Psychiatry. 2021 Jul 13;12:695017. doi: 10.3389/fpsyt.2021.695017 (PMC8313758; doi:10.3389/fpsyt.2021.695017)
Supplement: Supplementary file 1 [file Data_Sheet_1.docx]

**Supplementary Online Content**

**eTable 1.** Summary of first three parts of the questionnaire that was administered in this study.

**eTable 2.** Univariate regression analysis of risk factors associated with mental health symptoms and suicidal behavior.

**eTable1. Summary of first three parts of the questionnaire that was administered in this study.**

| **Question** | **Type** | **Choices** |
| --- | --- | --- |
| **Part One: Demographic information** | | |
| **Please fill in your university:** | Blank | / |
| **Please select your highest level of education:** | Single choice | Vocational school/College diploma/Bachelor’s degree/Master’s degree/Doctoral degree |
| **Please select your gender:** | Single choice | Male/Female |
| **Please fill in your date of birth:** | Blank | / |
| **Please fill in your height and weight:** | Blank | / |
| **Please select your nationality:** | Single choice | Han/National minority |
| **Please fill in your permanent geographical region:** | Blank | / |
| **Please select the marital status of your parent:** | Single choice | Married/Separated/Divorced/Widowed/Other |
| **Please select your monthly family**  **income (yuan):** | Single choice | <5000/5000-10000/10000-20000/20000-40000/≥40000 |
| **Are you satisfied with your major?** | Enter a number from 0 (not satisfied at all) to 10 (extremely satisfied) | / |
| **Are you satisfied with your academic performance?** | Enter a number from 0 (not satisfied at all) to 10 (extremely satisfied) | / |
| **How do you get along with your classmates?** | Single choice | Strained relationship/General relationship/ Harmonious relationship |
| **How do you get along with your teachers?** | Single choice | Strained relationship/General relationship/ Harmonious relationship |
| **Please select your level of academic performance in the class in the last half year** | Single choice | Top 10%/10-20%/20-50%/50-80%/80-100% |
| **Are you a graduate student?** | Single choice | Yes/No |
| **Are you back to school now?** | Single choice | Yes/No |
| **How do you get along with your father?** | Single choice | Distant relationship/General relationship/Close relationship |
| **How do you get along with your mother?** | Single choice | Distant relationship/General relationship/Close relationship |
| **Part Two: COVID-19 pandemic-related questions** | | |
| **Do you have any of the following conditions during the pandemic?** | Multiple choice | Confirmed COVID-19 case/Suspected COVID-19 case/Close contact with confirmed case/Participation in rescue services related to the pandemic/No |
| **Does your family have any of the following conditions during the pandemic?** | Multiple choice | Confirmed COVID-19 case/Suspected COVID-19 case/Close contact with confirmed case/Participation in rescue services related to the pandemic/No |
| **Have you experienced** **quarantine?** | Single choice | Home quarantine/Centralized quarantine/No quarantine/Other |
| **Please select your level of stress during the pandemic:** | Enter a number from 0 (not stressful at all) to 10 (extremely stressful) | / |
| **Do you have some changes in lifestyle during the pandemic?** | Single choice | Yes/No |
| **Have you learned some mental health information or received mental health intervention?** | Multiple choice | I have learned mental health information/I have received mental intervention/No |
| **Part Three: Behavioral habits** | | |
| **Have you ever used alcohol during the pandemic?** | Single choice | Yes/No |
| **Have you ever used tobacco during the pandemic?** | Single choice | Yes/No |
| **Do you engage in regular physical exercise during the pandemic?** | Single choice | Yes/No |

**eTable 2. Univariate regression analysis of risk factors associated with mental health symptoms and** **suicidal behavior.**

|  | **Depression^a^** | | **Anxiety^b^** | | **Insomnia^c^** | | **PTSD^d^** | | **Suicidal behavior** | |
| --- | --- | --- | --- | --- | --- | --- | --- | --- | --- | --- |
| **Variable** | OR (95% CI) | *p* value | OR (95% CI) | *p* value | OR (95% CI) | *p* value | OR (95% CI) | *p* value | OR (95% CI) | *p* value |
| Gender (ref: male) | | | | | | | | | | |
| Female | 1.15 (1.06-1.25) | < 0.01 | 1.10 (1.01-1.19) | 0.03 | 1.01 (0.93-1.09) | 0.87 | 0.72 (0.63-0.82) | < 0.01 | 1.72 (1.27-2.32) | < 0.01 |
| Are you graduates? (ref: no) | | | | | | | | | | |
| Yes | 1.14 (0.93-1.39) | 0.20 | 1.15 (0.93-1.41) | 0.19 | 1.27 (1.04-1.56) | 0.02 | 2.25 (1.72-2.95) | < 0.01 | 2.18 (1.32-3.61) | < 0.01 |
| Relationship with classmates and teachers (ref: harmonious) | | | | | | | | | | |
| Strained | 4.71 (2.40-9.24) | < 0.01 | 3.61 (1.94-6.70) | < 0.01 | 5.86 (2.98-11.51) | < 0.01 | 7.64 (3.98-14.69) | < 0.01 | 11.24 (4.62-27.33) | < 0.01 |
| General | 2.07 (1.92-2.24) | < 0.01 | 1.97 (1.82-2.13) | < 0.01 | 1.80 (1.66-1.94) | < 0.01 | 1.84 (1.61-2.11) | < 0.01 | 1.78 (1.36-2.33) | < 0.01 |
| Intimacy with parents (ref: close) | | | | | | | | | | |
| Distant | 4.81 (3.28-7.05) | < 0.01 | 4.84 (3.38-6.93) | < 0.01 | 4.45 (3.10-6.38) | < 0.01 | 7.48 (5.11-10.95) | < 0.01 | 13.57 (7.81-23.59) | < 0.01 |
| General | 2.39 (2.20-2.60) | < 0.01 | 2.31 (2.12-2.51) | < 0.01 | 2.06 (1.90-2.24) | < 0.01 | 2.72 (2.37-3.12) | < 0.01 | 3.68 (2.78-4.86) | < 0.01 |
| Positive individual history of mental health problem (ref: no) | | | | | | | | | | |
| Yes | 4.43 (4.04-4.85) | < 0.01 | 4.16 (3.81-4.55) | < 0.01 | 4.30 (3.93-4.70) | < 0.01 | 4.66 (4.06-5.34) | < 0.01 | 5.08 (3.87-6.66) | < 0.01 |
| Positive family history of mental health problem (ref: no) | | | | | | | | | | |
| Yes | 2.35 (1.91-2.90) | < 0.01 | 2.65 (2.15-3.26) | < 0.01 | 1.91 (1.56-2.35) | < 0.01 | 3.75 (2.90-4.86) | < 0.01 | 2.78 (1.66-4.63) | < 0.01 |
| Unknown | 2.34 (2.10-2.61) | < 0.01 | 2.17 (1.95-2.42) | < 0.01 | 1.78 (1.60-1.98) | < 0.01 | 2.42 (2.06-2.83) | < 0.01 | 2.16 (1.59-2.94) | < 0.01 |
| Are you infected with COVID-19 (confirmed or suspected case or close contact)? (ref: no) | | | | | | | | | | |
| Yes | 3.72 (2.25-6.16) | < 0.01 | 4.27 (2.64-6.90) | < 0.01 | 3.60 (2.24-5.80) | < 0.01 | 6.47 (4.04-10.37) | < 0.01 | 3.44 (1.38-8.61) | < 0.01 |
| Stress during COVID-19 pandemic (ref: low) | | | | | | | | | | |
| High | 4.44 (3.99-4.93) | < 0.01 | 4.87 (4.36-5.43) | < 0.01 | 3.84 (3.45-4.27) | < 0.01 | 6.08 (5.08-7.27) | < 0.01 | 9.88 (6.57-14.87) | < 0.01 |
| Moderate | 2.59 (2.37-2.82) | < 0.01 | 2.83 (2.57-3.11) | < 0.01 | 2.32 (2.12-2.54) | < 0.01 | 2.25 (1.87-2.70) | < 0.01 | 3.38 (2.20-5.19) | < 0.01 |
| Changes in lifestyle during the pandemic? (ref: no) | | | | | | | | | | |
| Yes | 2.37 (2.17-2.60) | < 0.01 | 2.26 (2.05-2.50) | < 0.01 | 2.37 (2.15-2.61) | < 0.01 | 2.02 (1.69-2.42) | < 0.01 | 2.52 (1.70-3.72) | < 0.01 |
| Alcohol use during COVID-19 pandemic (ref: no) | | | | | | | | | | |
| Yes | 1.70 (1.45-1.99) | < 0.01 | 1.59 (1.35-1.86) | < 0.01 | 1.66 (1.42-1.95) | < 0.01 | 2.30 (1.86-2.86) | < 0.01 | 2.99 (2.07-4.32) | < 0.01 |
| Tobacco use during COVID-19 pandemic (ref: no) | | | | | | | | | | |
| Yes | 1.51 (1.25-1.84) | < 0.01 | 1.55 (1.28-1.89) | < 0.01 | 1.43 (1.18-1.74) | < 0.01 | 2.44 (1.88-3.16) | < 0.01 | 1.96 (1.16-3.28) | 0.01 |
| Have you learned about mental health information during COVID-19 pandemic? (ref: no) | | | | | | | | | | |
| Yes | 0.91 (0.84-0.98) | 0.02 | 0.92 (0.85-1.00) | 0.04 | 0.92 (0.85-0.99) | 0.03 | 0.78 (0.68-0.89) | < 0.01 | 0.88 (0.67-1.14) | 0.32 |
| Do you engage in regular physical exercise during COVID-19 pandemic? (ref: no) | | | | | | | | | | |
| Yes | 0.66 (0.61-0.71) | < 0.01 | 0.70 (0.64-0.76) | < 0.01 | 0.67 (0.62-0.72) | < 0.01 | 0.72 (0.63-0.83) | < 0.01 | 0.81 (0.61-1.06) | 0.12 |
| Symptoms of depression (ref: no) | | | | | | | | | | |
| Yes | — |  | 41.46 (36.65-46.90) | < 0.01 | 9.78 (8.94-10.71) | < 0.01 | 51.87 (36.18-74.35) | < 0.01 | 10.52 (7.07-15.65) | < 0.01 |
| Symptoms of anxiety (ref: no) | | | | | | | | | | |
| Yes | 41.46 (36.65-46.90) | < 0.01 | — |  | 8.32 (7.61-9.10) | < 0.01 | 64.19 (46.45-88.71) | < 0.01 | 8.61 (6.22-11.93) | < 0.01 |
| Symptoms of insomnia (ref: no) | | | | | | | | | | |
| Yes | 9.78 (8.94-10.71) | < 0.01 | 8.32 (7.61-9.10) | < 0.01 | — |  | 18.20 (14.83-22.33) | < 0.01 | 5.06 (3.78-6.79) | < 0.01 |
| Symptoms of PTSD (ref: no) | | | | | | | | | | |
| Yes | 51.87 (36.18-74.35) | < 0.01 | 64.19 (46.45-88.71) | < 0.01 | 18.20 (14.83-22.33) | < 0.01 | — |  | 9.80 (7.48-12.83) | < 0.01 |
| Suicidal behavior (ref: no) | | | | | | | | | | |
| Yes | 10.52 (7.07-15.65) | < 0.01 | 8.61 (6.22-11.93) | < 0.01 | 5.06 (3.78-6.79) | < 0.01 | 9.80 (7.48-12.83) | < 0.01 | — |  |

^a^ Depression was defined as PHQ-9 score ≥ 5.

^b^ Anxiety was defined as GAD-7 score ≥ 5.

^c^ Insomnia was defined as ISI score ≥ 8.

^d^ PTSD was defined as PCL-5 score ≥ 33.
